# Hedgehog Inhibitors Beyond Clinical Complete Response in Basal Cell Carcinoma: Should I Stop or Should I Go?

**DOI:** 10.1093/oncolo/oyad319

**Published:** 2023-12-21

**Authors:** Salvatore Alfieri, Rebecca Romanò, Sara Marceglia, Vincenzo De Giorgi, Ketty Peris, Pietro Sollena, Alfredo Piccerillo, Ruggero Moro, Giulio Gualdi, Paolo Antonio Ascierto, Marco Palla, Miriam Paone, Laura Eibenschutz, Francesco Spagnolo, Paola Queirolo, Daria Maria Filippini, Stefano Cavalieri, Carlo Resteghini, Cristiana Bergamini, Antonello Manocchio, Lisa Licitra, Paolo Bossi

**Affiliations:** Head and Neck Medical Oncology Department, Fondazione IRCCS Istituto Nazionale dei Tumori, Milano, Italy; Head and Neck Medical Oncology Department, Fondazione IRCCS Istituto Nazionale dei Tumori, Milano, Italy; Department of Engineering and Architecture, University of Trieste, Trieste, Italy; Section of Dermatology, Department of Health Sciences, University of Florence, Firenze, Italy; UOC di Dermatologia, Dipartimento di Scienze Mediche e Chirurgiche Addominali ed Endocrino Metaboliche, Fondazione Policlinico Universitario A Gemelli - IRCCS, Roma, Italy; Dermatologia, Dipartimento di Medicina e Chirurgia Traslazionale, Università Cattolica del Sacro Cuore, Roma, Italy; UOC di Dermatologia, Dipartimento di Scienze Mediche e Chirurgiche Addominali ed Endocrino Metaboliche, Fondazione Policlinico Universitario A Gemelli - IRCCS, Roma, Italy; UOC di Dermatologia, Dipartimento di Scienze Mediche e Chirurgiche Addominali ed Endocrino Metaboliche, Fondazione Policlinico Universitario A Gemelli - IRCCS, Roma, Italy; Dermatologia, Dipartimento di Medicina e Chirurgia Traslazionale, Università Cattolica del Sacro Cuore, Roma, Italy; Escuela de Doctorado, Universidad Católica de Valencia San Vicente Martir, Valencia, Spain; Dermatologic Clinic, Department of Medicine and Aging Science, Università G d’Annunzio, Chieti-Pescara, Italy; Unit of Melanoma Cancer Immunotherapy and Innovative Therapy, National Tumour Institute IRCCS Fondazione G. Pascale, Napoli, Italy; Unit of Melanoma Cancer Immunotherapy and Innovative Therapy, National Tumour Institute IRCCS Fondazione G. Pascale, Napoli, Italy; Unit of Melanoma Cancer Immunotherapy and Innovative Therapy, National Tumour Institute IRCCS Fondazione G. Pascale, Napoli, Italy; Dermatologia Oncologica e Prevenzione, Istituto San Gallicano IRCCS, Roma, Italy; Medical Oncology 2, IRCCS Ospedale Policlinico San Martino, Genova, Italy; Department of Surgical Sciences and Integrated Diagnostics (DISC), Plastic Surgery Division, University of Genova, Genova, Italy; Istituto Europeo di Oncologia - IRCCS, Milan, Italy; Head and Neck Medical Oncology Department, Fondazione IRCCS Istituto Nazionale dei Tumori, Milano, Italy; Department of Oncology and Hemato-oncology, University of Milan, Milano, Italy; Head and Neck Medical Oncology Department, Fondazione IRCCS Istituto Nazionale dei Tumori, Milano, Italy; Head and Neck Medical Oncology Department, Fondazione IRCCS Istituto Nazionale dei Tumori, Milano, Italy; Head and Neck Medical Oncology Department, Fondazione IRCCS Istituto Nazionale dei Tumori, Milano, Italy; Department of Oncology and Hemato-oncology, University of Milan, Milano, Italy; Head and Neck Medical Oncology Department, Fondazione IRCCS Istituto Nazionale dei Tumori, Milano, Italy; Department of Oncology and Hemato-oncology, University of Milan, Milano, Italy; Head and Neck Medical Oncology Department, Fondazione IRCCS Istituto Nazionale dei Tumori, Milano, Italy; Medical Oncology Unit, ASST Spedali Civili di Brescia, Brescia, Italy; Department of Medical and Surgical Specialties, Radiological Sciences, and Public Health, University of Brescia, Brescia, Italy

**Keywords:** basal cell carcinoma, hedgehog inhibitors, vismodegib, sonidegib, beyond complete response, maintenance therapy

## Abstract

**Introduction:**

In advanced basal cell carcinoma (BCC), the issue of whether Hedgehog inhibitors (HHIs) should be stopped or not after clinical complete response (cCR) achievement remains an unmet clinical need.

**Materials and Methods:**

We conducted a retrospective, multicenter study across 7 Italian dermato-oncology units including patients with BCC who continued vismodegib after cCR between 2012 and 2019. We assessed the relationship between the duration of vismodegib intake (days to cCR [DTCR], days to stop after cCR [DTS], total treatment days [TTD]), and disease-free survival (DFS). Reasons to stop vismodegib were (R1) toxicity and (R2) disease recurrence. The relationship between DTCR, DTS, TTD, and DFS in the whole population and in R1 subgroup was assessed by Pearson’s correlation coefficient (*P* < .05) and Bayesian statistics (BF_10_).

**Results:**

Sixty-eight BCC patients with a median (m) age of 75.5 years (39-100) were included. Most patients were male (*N* = 43, 63%), without Gorlin syndrome (*N* = 56, 82%) and with head and neck area as primary site (*N* = 51, 75%). After cCR, out of 68 patients, 90% (*N* = 61/68) discontinued vismodegib: 82% (*N* = 50/61) due to toxicity (R1), and 18% (*N* = 11/61) due to recurrence (R2). Conversely, 10% (*N* = 7/68) continued vismodegib until last follow-up. In the whole population (*N* = 68), cCR was achieved with a mDTCR of 180.50 days. DFS showed a significant correlation with DTS (*P* < .01, BF_10_ = 39.2) and TTD (*P* < .01, BF_10_ = 35566), while it was not correlated to DTCR (BF_10_ < 0.1). The analysis of R1 subgroup (*N* = 50) confirmed these results. DFS correlated with DTS in all recurrent patients (*N* = 38, *r* = 0.44, *P* < .01) and in the recurrent patients who stopped vismodegib for toxicity (*N* = 26, *r* = 0.665, *P* < .01). DFS was longer when vismodegib was maintained for >2 months after cCR (mDFS > 2 months, *N* = 54 vs. ≤ 2 months, *N* = 14: 470 vs. 175 d, *P* < .01).

**Conclusions:**

Our retrospective results suggest that HHIs should be continued after cCR to improve DFS in BCC.

## Teaser

In advanced basal cell carcinoma, the issue of whether Hedgehog inhibitors should be stopped after clinical complete response remains an unmet clinical need. This study compared disease-related outcomes among patients who either discontinued or maintained the treatment after achievement of clinical complete response.

**Implications for Practice**

In advanced basal cell carcinoma (BCC), the issue of whether Hedgehog inhibitors (HHIs) should be stopped or not after clinical complete response (cCR) achievement remains an unmet clinical need. The authors carried out a retrospective, multicenter study to compare disease-related outcomes among patients with BCC who either discontinued or maintained HHIs treatment after cCR achievement. It was observed that a larger number of total days on treatment with vismodegib, as well as a longer duration of maintenance therapy after cCR, were directly correlated to better DFS in patients with laBCC and mBCC. No significant correlation was found between time to cCR and DFS, potentially highlighting the value of vismodegib maintenance therapy. This retrospective analysis supports the benefit of longer HHIs maintenance therapy beyond cCR in patients with BCC. Further larger, randomized, prospective studies will be needed to better establish whether to stop or to continue HHIs after cCR achievement.

## Introduction

Basal cell carcinoma (BCC) represents the most common type of non-melanoma skin cancer.^1-4^ The main molecular pathogenetic driver of BCC is constituted by the aberrant activation of the *Hedgehog (Hh)* pathway, involving mutations of *patched-1 (PTCH1)* and *smoothened (SMO)* genes.^5^ Moreover, Gorlin syndrome (GS), resulting from *PTCH1* germline mutations,^6-9^ is a well-established, hereditary BCC-predisposing disorder.

Although often curable through surgery or radiotherapy (RT), BCC may also evolve to locally advanced (la) or, very rarely, metastatic (m) stages.^10^ The call for appropriate therapeutic strategies for these settings, supported by the growing knowledge surrounding the *Hh* pathway, has led to the development of the oral Hedgehog inhibitors (HHIs),^6,11,12^ vismodegib,^13,14^ and sonidegib.^15,16^

Vismodegib, the first-in-class *SMO* inhibitor, was approved by the FDA (January 2012) and EMA (July 2013) for the treatment of laBCC not eligible for radical surgery/RT, as well as for mBCC.^13,14^ In phase II, ERIVANCE BCC trial,^17^ daily 150 mg achieved complete response (CR) rates of 32% (20/63) in laBCC and 0% (0/33) in mBCC, respectively.^18^ CTCAE grades 3-4 adverse events (AEs) occurred within a range of 23%-55%, leading to permanent drug discontinuation in 21% of cases.

The second HHI to receive FDA (July 2015) and EMA (August 2015) approval for use in patients with laBCC not amenable to curative surgery/RT was sonidegib.^15,16^ In phase II BOLT trial,^19^ CR rates for laBCC were 5% in the 200 mg group (3/66) and 1.6% (2/128) in the 800 mg group, respectively, while no CR was observed in mBCC.^20^ The 200 mg dose became the standard in clinical practice, being associated with a lower incidence of grades 3-4 AEs (32% vs. 43%); however, discontinuation rates were similar as compared to the 800 mg cohort (14% vs. 14.7%).

Given the frequency of AEs affecting treatment tolerability, the phase II MIKIE trial investigated two different, intermittent vismodegib dosing regimens. Despite the significant disease control outcomes, the overall discontinuation rate was 23% (53/229), as previously observed with continuous schedules.^17^

In lack of significant improvements in the management of HHIs toxicity profile, there arises the clinical dilemma of whether to stop or to maintain HHIs after CR achievement. Indeed, no data could be extrapolated from either ERIVANCE or BOLT trial, as patients continued HHIs either until disease progression (PD) or unacceptable toxicity.^17,19^ Moreover, Herms et al^21^ evaluated the long-term outcomes of 116 patients with laBCC who had achieved CR on vismodegib and subsequently discontinued treatment. However, as all included subjects discontinued vismodegib shortly after CR, no useful data could be extrapolated concerning HHIs treatment extension beyond CR.

Building from these premises, we have designed and carried out a retrospective, multicenter study, to compare disease-related outcomes among patients with BCC who either discontinued or maintained HHIs treatment after clinical complete response (cCR) achievement.

## Methods

### Study Design and Population

A retrospective, observational, multicenter study was conducted in 7 onco-dermatology units based in the following Institutions: Fondazione IRCCS Istituto Nazionale dei Tumori (Milano), Presidio Ospedaliero Piero Palagi (Firenze), Policlinico Universitario Agostino Gemelli (Roma), Spedali Civili (Brescia), Istituto Nazionale Tumori Regina Elena e Istituto Dermatologico San Gallicano (Roma), Istituto Nazionale Tumori IRCCS Fondazione Pascale (Napoli), Ospedale Policlinico San Martino (Genova).

The study was approved by the internal ethical committee of the coordinating center (N. INT 29/23) and by each independent ethical committee of the clinical institutions joining the study.

Patients with laBCC or mBCC, with or without Gorlin syndrome, who had been treated with vismodegib between March 2012 and January 2019, and who had achieved cCR, were included. Both cCR and disease relapse were assessed clinically (ie, without pathological confirmation), integrating medical imaging when deemed appropriate (eg, in case of distant metastasis or of deep local infiltration).

All patients were treated with vismodegib as, at the time of data collection, sonidegib had not yet been approved and reimbursed in Italy, nor was it available in any active clinical trials at the participating centers. Vismodegib was initiated either within the clinical trials STEVIE^22^ and MIKIE^23^ or after marketing authorization was obtained in Italy (March 23rd, 2015).

The following clinical data were collected: patients’ age, gender, diagnosis of Gorlin syndrome, clinical disease stage (laBCC vs. mBCC), and primary tumor site (eg, head/neck, limbs, trunk). The time of vismodegib initiation, cCR achievement, and treatment discontinuation was noted, as well as the causes for discontinuation. During follow-up, the date of disease relapse was registered, while we did not include toxicity data in the final analysis.

### Drug Intake

Vismodegib was given at a daily dose of 150 mg. Patients who experienced CTCAE grade 3 or 4 AEs, after drug discontinuation to reverse toxicity, were then managed as per local clinical protocols, in accordance with specific guidelines.^24^

To assess the relationship between the duration of vismodegib intake and disease-related outcomes, the following time points were analyzed: the number of days of treatment needed to achieve cCR (DTCR), days to vismodegib stop after cCR (DTS), total treatment days (TTD = DTCR + DTS). Disease-free survival (DFS) was defined as the number of days from cCR to recurrence (or to last follow-up in those patients who did not experience disease recurrence). Reasons to stop vismodegib were classified as (R1) toxicity and (R2) disease recurrence. Concerning the R1 group, patients’/physicians’ decision to withdraw the treatment was usually due to unacceptable correlated symptoms and/or intolerable impact on patients’ quality of life; therefore, we assimilated patients’/physician’s choice and toxicity within a single variable.

### Study Objectives

Our objective was to evaluate the impact of vismodegib maintenance therapy after cCR on disease-related outcomes and, specifically, to assess whether a prolonged vismodegib intake after cCR achievement was associated with better DFS.

### Statistical Analysis

In this retrospective evaluation, we first characterized the population considering age, gender, the presence of Gorlin syndrome, and the location of the primary tumor. Descriptive statistics were used at this stage. We then proceeded to univariate analysis using nonparametric statistics. The effect of categorical variables was verified using the Wilcoxon signed rank test (*P* < .05). The possible relationship between continuous variables was assessed using correlation analysis with Spearman’s ρ correlation coefficient.

Since this was a retrospective analysis aimed to highlight the possible effects of prolonged vismodegib intake over disease control in BCC, we analyzed DFS more in depth. More specifically, factors significantly influencing DFS in the univariate analysis were included in a multivariate analysis to verify their concurrent effect.

In addition, we further analyzed the effects on DFS using Bayesian statistics, as suggested by recent literature.^25^ The Bayesian statistical approach is based on the estimation of the Bayes factor (BF_10_) that represents the ratio between the likelihood of the alternative hypothesis to explain the observed data and that of the null hypothesis. This allows to estimate both the evidence in favor of the alternative hypothesis (BF_10_ > 1) and that in favor of the null hypothesis (BF_10_ < 1), and therefore to support not only the existence of an effect but also the absence of an effect. Standard evidence levels for the one-tailed BF_10_ were used: anecdotal (1/3 < BF_10_ < 3), moderate (<1/3 or >3), strong (<1/10 or >10), very strong (<1/30 or >30), and decisive (<1/100 or >100). The JASP software (version 0.16.3, University of Amsterdam, NL) was used for the analysis.^25^

## Results

### Patients’ Clinical Characteristics and Correlation With Treatment Duration

Out of a total of 227 patients treated with vismodegib among the 7 participating centers, 68 patients (30%) affected by laBCC (*N* = 65, 96%) and mBCC (*N* = 3, 4%) who achieved cCR were included in the analyses. The median age was 75.5 years old (range 39-100) with a male gender prevalence (43 males, 63% vs. 25 females, 37%). Twelve patients (18%) were affected by Gorlin syndrome. Head and neck area was the primary site of BCC lesions in most patients (*N* = 51, 75%). During the treatment course, 9/68 (13%) patients received alternative dosing schedules, while 7/68 (10%) patients received additional treatments (eg, surgery, radiotherapy, and topical treatment) due to oligo-progression of disease. The main clinical characteristics of study population are summarized in Table 1.

**Table 1.** Clinical characteristics of study population (*N* = 68).

Age	Mean (SD)	72.99 (15.84)
	Median (range)	75.50 (39-100)
Gender	Male (%)	43 (63%)
	Female (%)	25 (37%)
Gorlin syndrome	Yes (%)	12 (18%)
	No (%)	56 (82%)
Disease site	Head and neck (%)	51 (75%)
	Upper/lower limbs (%)	2 (3%)
	Trunk (%)	12 (18%)
	Upper/lower limbs and trunk (%)	3 (4%)
Stage	Locally advanced (%)	65 (96%)
	Metastatic (%)	3 (4%)
Disease recurrence (*N* = 61)^*^	Yes (%)	38 (62%)
	No (%)	23 (38%)
Patient status by the end of follow-up	Alive (%)	49 (72%)
	Dead for BCC (%)	6 (9%)
	Dead for other causes (%)	13 (19%)

^*^Applicable to the 61 patients who discontinued vismodegib (7 patients received vismodegib until last follow-up).

At a median follow-up of 42.5 months (range 0-91), cCR was achieved with a median (m)DTCR of 180.50 days, with no differences related to age or gender. Neither Gorlin syndrome nor the location of the primary disease were related to DTCR. Details are provided in Table 2.

**Table 2.** Correlation between treatment duration and patients’ clinical characteristics.

Days to complete response (DTCR)			*P*-value
Total population (*N* = 68)	Mean (SD) Median (range)	205.12 (101.66) 180.50 (56-595)	
Gender	Male (*N* = 43) Female (*N* = 25)	188.0 (56-595) 176.0 (86-344)	.67
Age	Above median (>75 years) (*N* = 34) Below median (≤75 years) (*N* = 34)	177.0 (60-280) 205.5 (56-595)	.30
Gorlin syndrome	Yes (*N* = 12) No (*N* = 56)	226.0 (87-595) 175.0 (56-562)	.06
Disease site	Head and neck (*N* = 51) Other (*N* = 17)	178.0 (60-595) 221.0 (56-506)	.53
Days to vismodegib stop after cCR (DTS)			
Total population (*N* = 61)^*^	Mean (SD) Median (range)	221.3 (226.7) 125.0 (0-1018)	
Gender	Male (*N* = 39) Female (*N* = 22)	139.0 (0-1018) 119.0 (29-920)	.46
Age	Above median (>75 years) (*N* = 33) Below median (≤75 years) (*N* = 28)	119.0 (0-920) 150.5 (26-1018)	.44
Gorlin syndrome	Yes (*N* = 9) No (*N* = 52)	125.0 (69-697) 127.0 (0-1018)	.90
Disease site	Head and neck (*N* = 47) Other (*N* = 14)	119.0 (26-1018) 199.5 (0-876)	.67
Total treatment days (TTD)			
Total population (*N* = 68)	Mean (SD) Median (range)	530.6 (411.8) 403.5 (95-2112)	
Gender	Male (*N* = 43) Female (*N* = 25)	446.0 (95-1530) 317.0 (147-2112)	.25
Age	Above median (>75 years) (*N* = 34) Below median (≤75 years) (*N* = 34)	320.5 (95-2112) 513.0 (147-2009)	**.03** ^**^
Gorlin	Yes (*N* = 12) No (*N* = 56)	548.0 (207-2009) 371.5 (95-2112)	.09
Disease site	Head and neck (*N* = 51) Other (*N* = 17)	371.5 (147-2112) 503.0 (95-2009)	.52

^*^Applicable to the 61 patients who discontinued vismodegib (7 patients received vismodegib until last follow-up).

^**^ *P* < .05 is considered significant. Values are reported in median (range).

Concerning DTS, the overall mDTS was 125 days, with no differences due to patient age or gender, Gorlin syndrome, and primary lesion subsite (Table 2).

Regarding TTD, the overall mTTD was 403.5 days. Patients below the median age of 75 years had significantly longer treatment duration (≤75 years old: 512.0 days; >75 years old: 321.0 days, *P* = .03). Patient gender, Gorlin syndrome, and the primary disease subsite had no effect on TTD (Table 2).

### Treatment Discontinuation

After cCR, 61 patients (90%) discontinued vismodegib, while 7 patients (10%) continued to receive vismodegib until last follow-up, resulting in a statistically significant longer total treatment course (mTTD: 1530 days vs. 367 days, *P* < .01; Supplementary Table S1). With a mDTS of 125 days, 54 patients (79%) received vismodegib for >2 months, and the other 14 (21%) received vismodegib for ≤2 months after cCR. Vismodegib discontinuation after cCR was mostly due to toxicity (*N* = 50, 82%) while 11 patients (18%) discontinued due to disease recurrence. Moreover, mDTS and mTTD were significantly longer in patients who stopped vismodegib for recurrence with respect to patients who stopped it for toxicity (Supplementary Table S1).

### Disease Recurrence

Disease recurrence after cCR was observed in 38 (62.3%) patients after a median of 357 days. In all cases, disease recurrence occurred with a loco-regional pattern. Patients who experienced disease recurrence had a significantly longer mDTCR with respect to those who did not recur (mDTCR: 222.5 vs. 168 days, *P* = .03). In the same setting, there was no significant difference observed neither in mDTS (192 vs. 85.5 days, *P* = .05) nor in mTTD (497.5 vs. 319 days, *P* = .32). After recurrence, 35/38 (92%) patients received post-relapse treatments: in detail, 11 patients received surgery, 3 patients received radiotherapy, 1 patient received chemotherapy, 10 patients received a topical treatment (eg, laser therapy, cryotherapy, and photodynamic therapy), and 10 patients received a combination among at least two of the above-described treatment modalities. Furthermore, 7/50 (14%) patients who discontinued vismodegib due to toxicity were rechallenged with HHI: 4 patients showed partial response, while 3 patients achieved disease stability as best response. The correlation between days to vismodegib stop (DTS) and days from vismodegib stop to disease recurrence (ie, DFS-DTS) was depicted in Supplementary Figure 1.

### Correlation Between DFS, Patients’ Clinical Characteristics, Treatment Discontinuation, and Disease Recurrence

The mDFS was 414.5 days, and it was not influenced by patient age, gender, Gorlin disease (mDFS Gorlin: 637.7 days vs. mDFS non-Gorlin: 633.1 days; *P* = .90), or primary disease subsite (Table 3). Of interest, although not statistically significant, 9/12 (75%) of Gorlin patients experienced disease recurrence, as compared with 25/56 (45%) of patients with sporadic BCC. However, time from cCR to disease recurrence was overall comparable between the two subgroups, with a slight tendency towards a slower recurrence among patients with Gorlin (mean time to recurrence: Gorlin: 536.9 days vs. non-Gorlin: 462 days; *P* = .60).

**Table 3.** Correlation between DFS, patients’ clinical characteristics, treatment discontinuation, and disease recurrence.

		Disease-free survival (DFS)	*P*-value	Bayes Factor BF_10_
Total population (*N* = 68)	Mean (SD)	633.9 (577.5)		
	Median (range)	414.5 (0-2600)		
Gender	Male (*N* = 43)	465.0 (0-2077)	.30	0.29
	Female (*N* = 25)	323.0 (29-2600)		
Age	Above median (>75 years)(*N* = 34)	336.0 (0-2600)	.20	0.18
	Below median (≤75 years)(*N* = 34)	502.0 (29-2077)		
Gorlin syndrome	Yes (*N* = 12)	431.5 (117-1788)	.69	0.31
	No (*N* = 56)	414.5 (0-2600)		
Disease site	Head and neck (*N* = 51)	449.0 (29-2600)	.35	0.78
	Other (*N* = 17)	349.0 (0-1788)		
Disease recurrence after cCR	Yes (*N* = 38)	357.0 (60-1792)	.13	**7.86**
	No (*N* = 30)	712.0 (0-2600)		
Vismodegib discontinuation	Yes (*N* = 61)	365.0 (0-2600)	**.01** ^**^	**23.26**
	No (*N* = 7)	1261.0 (182-1955)		
Reason for vismodegib discontinuation (*N* = 61)^*^	Toxicity (*N* = 50)	404.5 (0-2600)	.11	2.35
	Recurrence (*N* = 11)	322.0 (60-479)		

^*^Applicable to the 61 patients who discontinued vismodegib (7 patients received vismodegib until last follow-up).

^**^ *P* < 0.05 is considered significant. Values are reported in median (range). Statistically significant values (including moderate-to-strong and decisive evidence as per Bayesian analysis) are reported in bold.

Regardless of the reason for discontinuation, patients who continued vismodegib had a significantly longer DFS than those who discontinued (mDFS discontinuing vismodegib: 365.0 days vs. mDFS continuing vismodegib: 1261.0 days, *P* = .01; Table 3). This is in line with the observation that patients receiving vismodegib for >2 months after cCR had a significantly longer DFS than those discontinuing the treatment within 2 months after cCR (mDFS > 2 months: 470 days vs. mDFS ≤ 2 months: 175 days, *P* = .01).

The correlation between DFS and disease recurrence after cCR did not reach statistical significance, although it did show a positive trend (mDFS in patients recurring after cCR: 357.0 days vs. mDFS in patients not recurring after cCR: 712.0 days, *P* = .13). As seen in Table 3, Bayesian analysis highlighted that non-recurring patients had moderate-to-strong evidence of increased DFS as compared to recurring patients (BF_10_ = 7.861).

### Correlation Between DFS and Treatment Duration

**Figure 1.**
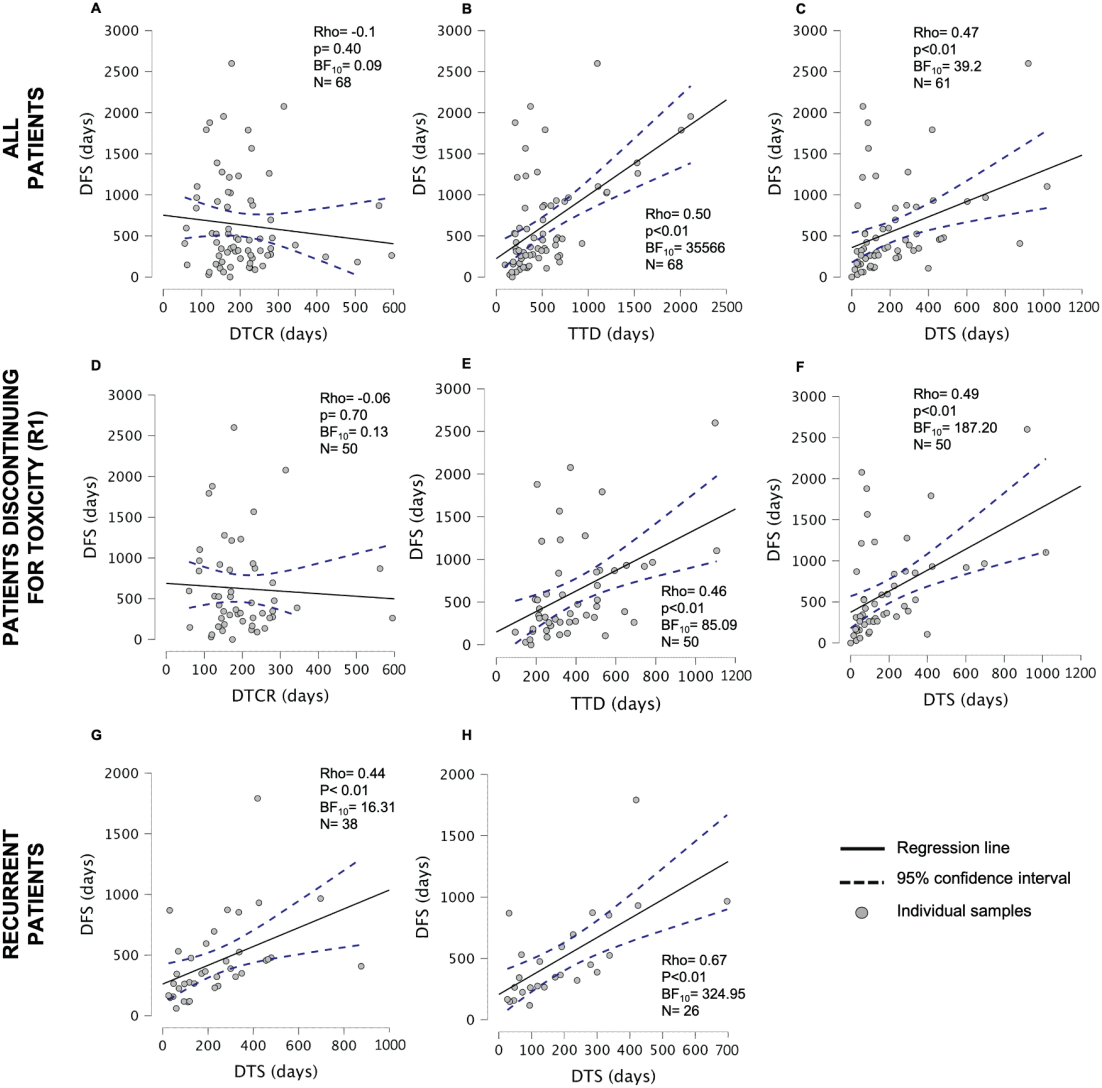
Top row: The correlation between treatment days and disease-free survival (DFS) in the whole population. Each plot represents the estimated correlation between DFS and days to complete response (DTCR, panel **A**, *N* = 68), total treatment days (TTD, panel **B**, *N* = 68), and days to vismodegib stop (DTS, panel **C**, *N* = 61 because 7 patients did not stop vismodegib). Central row. The correlation between treatment duration and DFS in the R1 subgroup (*N* = 50 patients discontinuing vismodegib for toxicity). Each plot represents the estimated correlation between DFS and DTCR (panel **D**), TTD (panel **E**), and DTS (panel **F**). Bottom row. The correlation between DTS and DFS in all recurrent patients (*N* = 38, panel **G**) and in patients who recurred after discontinuing vismodegib for toxicity (*N* = 26, panel **H**). Each plot represents individual patient values (grey circles), estimated linear regression slope (solid line), and the 95% confidence intervals (dashed lines).

DFS was not correlated to DTCR, thus suggesting, with strong evidence, that the number of days before cCR did not influence the number of days before recurrence or last follow-up (Spearman’s ρ = −0.1, *P* = .41, BF_10_ = 0.09, Figure 1, top row). Conversely, DFS was significantly correlated to TTD (Spearman’s ρ = 0.50, *P* < .01, Figure 1, top row), with Bayesian approach showing decisive evidence (BF_10_ = 35566). Similarly, in patients who discontinued vismodegib (*N* = 61), DFS correlated with DTS (Spearman’s ρ = 0.47, *P* < .01, Figure 1, top row) with decisive evidence (BF_10_ = 39.2).

Multivariate analysis including TTD, recurrence, and vismodegib discontinuation showed that all these three variables significantly influenced DFS (overall model *P* < .01; TTD *P* < .01; vismodegib discontinuation, *P* = .02; recurrence, *P* = .01).

Since the whole population included also patients discontinuing vismodegib for recurrence (ie, for whom DFS = DTS), possibly biasing the results of the correlation, we repeated the analysis considering only patients who discontinued vismodegib for toxicity (R1, *N* = 50) and obtained the same results (Figure 1, middle row): DFS had a significant direct correlation with both DTS (*r* = 0.49, *P* < .01, BF_10_ = 187.20) and TTD (*r* = 0.46, *P* < .01, BF_10_ = 85.10), but not with DTCR (r = −0.06, *P* = .70, BF_10_ = 0.13).

In recurrent patients, DFS was positively correlated with DTS (*r* = 0.44, *P* < .01, Figure 1, bottom row), thus suggesting that the longer was vismodegib intake after cCR, the longer the time to disease recurrence. This was confirmed also in the 26 recurrent patients who stopped vismodegib for toxicity (*r* = 0.67, *P* < .01, Figure 1, bottom row).

**Figure 2.**
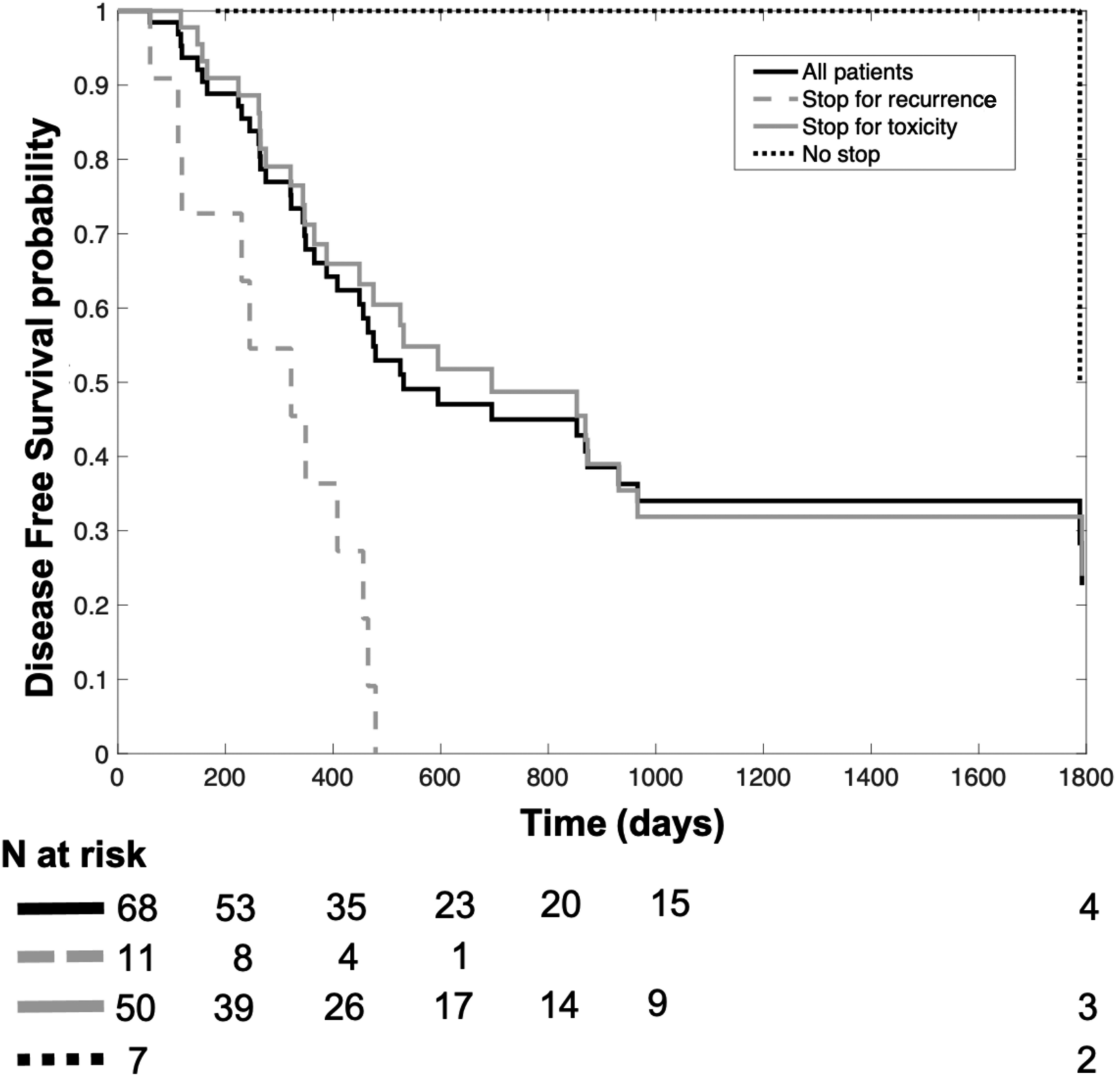
Kaplan-Meier plot of the cumulative probability function of DFS in the whole population (black solid line, *N* = 68), the R2 subgroup (grey dashed line, *N* = 11 patients discontinuing vismodegib for recurrence), the R1 subgroup (grey solid line, *N* = 50 patients discontinuing vismodegib for toxicity), and the subgroup of patients who continued vismodegib (black dotted line, *N* = 7). The subgroup of patients who continued vismodegib includes one patient who maintained treatment beyond minimal PD (recurrence observed after 1788 days from CR), causing the drop of probability from 1 to 0.5. Considering only patients responsive to vismodegib (ie, excluding the primary resistant R2 subgroup), the most relevant benefit is seen for patients who continued vismodegib (black dotted line), while patients discontinuing vismodegib for toxicity (grey solid line) followed a similar trajectory to R2 patients (grey dashed line), further stressing the negative impact of vismodegib discontinuation on DFS. Note that the whole population (black solid line) followed a similar trajectory to patients discontinuing vismodegib for toxicity (grey solid line), which is in line with the higher representation of the R1 subgroup in our sample (*N* = 50/68).

Figure 2 portrays the Kaplan-Maier curves for DFS of (a) all patients (*N* = 68), as well as those of the three subgroups of interest, respectively; (b) patients who continued vismodegib after cCR (*N* = 7); (c) patients who discontinued vismodegib for toxicity, ie, R1 subgroup (*N* = 50); (d) patients who discontinued vismodegib for recurrence, ie, R2 subgroup (*N* = 11). As shown, the curves of R1 and R2 subgroups share a similar trajectory: in other words, the disease outcomes of patients who discontinued vismodegib due to toxicity were not exceedingly better as compared to those of recurring patients.

## Discussion

In our study, we observed that a larger number of total days on treatment with vismodegib, as well as a longer duration of maintenance therapy after cCR, were directly correlated to better DFS in patients with laBCC and mBCC. Such association was seen in all-comers, as well as in patients who experienced recurrence, maintaining statistical significance also when considering only subjects who discontinued vismodegib due to toxicity. Moreover, no significant correlation was found between time to cCR and DFS, potentially highlighting the value of vismodegib maintenance therapy in this regard.

While the prospective SONIBEC trial is currently ongoing (NCT04806646) with the aim of investigating a tailored, intermittent sonidegib schedule after cCR in laBCC, our retrospective, multicentric sample is the largest one addressing the yet unsolved clinical dilemma of how to manage HHIs after cCR achievement. In this respect, one previous Italian, retrospective study^26^ reported data concerning the long-term efficacy of low-dose vismodegib maintenance therapy after cCR in 42 patients with laBCC: at 1-year follow-up, no relapse was observed among patients in the maintenance arm, while 26.6% (4/15) experienced recurrence after stopping vismodegib, also suggesting that a longer treatment exposure can improve oncologic outcomes, in line with previous reports.^27^

Our results are supported by previous literature documenting the pivotal role of the aberrantly activated *Hh* pathway in BCC development and progression.^17,18,21,22,28,29^ Moreover, analogous encouraging data can be derived across different, oncogene-addicted cancers, where the prolonged use of molecular-driven targeted agents — eg, beyond PD — has provided significant clinical benefits.^30-34^ In the light of the significant correlation between TTD and DFS, it seems plausible to assume that extending HHIs treatment beyond cCR could act as a brake against BCC recurrence. Although immunotherapy has recently emerged as a novel therapeutic option after HHIs failure, the observed results do not seem to always yield remarkable response rates, especially if compared to other more immunogenic non-melanoma skin cancers (ie, cutaneous squamous cell carcinoma and Merkel cell carcinoma^35^); therefore, notwithstanding this valid alternative, to date HHIs still stand as the best upfront systemic treatment option for laBCC and mBCC and should be exploited for as long as feasible.^36,37^

On the other hand, HHIs’ tolerability profile poses relevant practical challenges: in our study, most patients discontinued vismodegib during follow-up (61/68), with shorter DFS in patients stopping either for recurrence or, more often, for toxicity (Figure 2); also, as expected, older (>75 years old) subjects experienced shorter TTD (Table 2).

While attempts have been made to limit HHIs toxicities, and alternative treatment schedules (ie, intermittent dosing) have been explored,^23^ to date, the tools for an efficacious management of HHIs side effects are still limited. Further studies are awaited to provide clearer guidance on the best approach to HHIs treatment doses, schedules, and duration, balancing toxicity on one side and clinical benefits on the other. This may be especially relevant for HHIs maintenance therapy in patients who achieve a later cCR, who presumably embody a more biologically aggressive and resistant disease subset: in this context, prolonging HHIs molecular brake, while managing concurrent toxicities for as long as possible, may prove particularly beneficial to further delay recurrence.

As far as limitations are concerned, in our retrospective and limited sample we only included vismodegib for the reasons mentioned in the Introduction. However, despite the lack of a head-to-head comparison, sonidegib and vismodegib demonstrated similar efficacy and safety profiles from the pivotal BOLT^19^ and ERIVANCE^17^ trials, respectively: therefore, our results may be reasonably extended to sonidegib.^38-40^ Moreover, we only evaluated cCR clinically, without histological confirmation and without a baseline measurement of tumor size: although this may represent a source of bias, in routine medical practice BCC response and relapse are chiefly clinically determined. Also, we assumed that patient’s/physician’s preference could be assimilated to unacceptable toxicity when evaluating the reasons for vismodegib discontinuation: while acknowledging that this may affect the precision of our results, this appeared like a reasonable approximation. Furthermore, we did not include toxicity data in the final analysis; however, this was beyond the scope of our study, as similar data can be extrapolated from the BOLT^19^ and ERIVANCE^17^ trials, as well as from Scalvenzi et al.^26^ In addition, our study population was characterized by a fairly high prevalence of metastatic patients (4% mBCC in our sample): this is likely the result of a selection bias, as our work necessarily included only patients with BCC who required an active oncological treatment. Last, given the retrospective nature of our work, we chose to focus on DFS to provide a straight-forward, practical measure of oncologic outcome. Indeed, most patients with BCC usually have an excellent long-term prognosis, which is not significantly impacted by their oncological disease, hence long-term OS-related outcomes were beyond our scopes. In this respect, the lack of a longer-term follow-up prevented the collection of any data concerning vismodegib activity in terms of cancer interception, in particular within Gorlin population.^28^

## Conclusion

This retrospective analysis supports the benefit deriving from a longer HHIs maintenance therapy beyond cCR in patients with BCC, while always considering treatment tolerability. Further larger, randomized, prospective studies will be needed to better establish whether to stop or to continue HHIs after cCR achievement.

## Supplementary Material

Supplementary material is available at *The Oncologist* online.

oyad319_suppl_Supplementary_Figure

oyad319_suppl_Supplementary_Table

## Data Availability

The data underlying this article will be shared on reasonable request to the corresponding author.
