# Supplementary material for: Hedgehog Inhibitors Beyond Clinical Complete Response in Basal Cell Carcinoma: Should I Stop or Should I Go?
Source: Oncologist. 2023 Dec 21;29(5):e699–707. doi: 10.1093/oncolo/oyad319 (PMC11067794; doi:10.1093/oncolo/oyad319)

**Supplementary Figure 1** – The correlation between days to vismodegib stop (DTS) and days from vismodegib stop to disease recurrence (i.e., DFS-DTS) in the subgroup of patients who recurred after discontinuing vismodegib for toxicity (N=26). The plot represents individual patient values (grey circles), estimated linear regression slope (solid line) and the 95% confidence intervals (dashed lines).


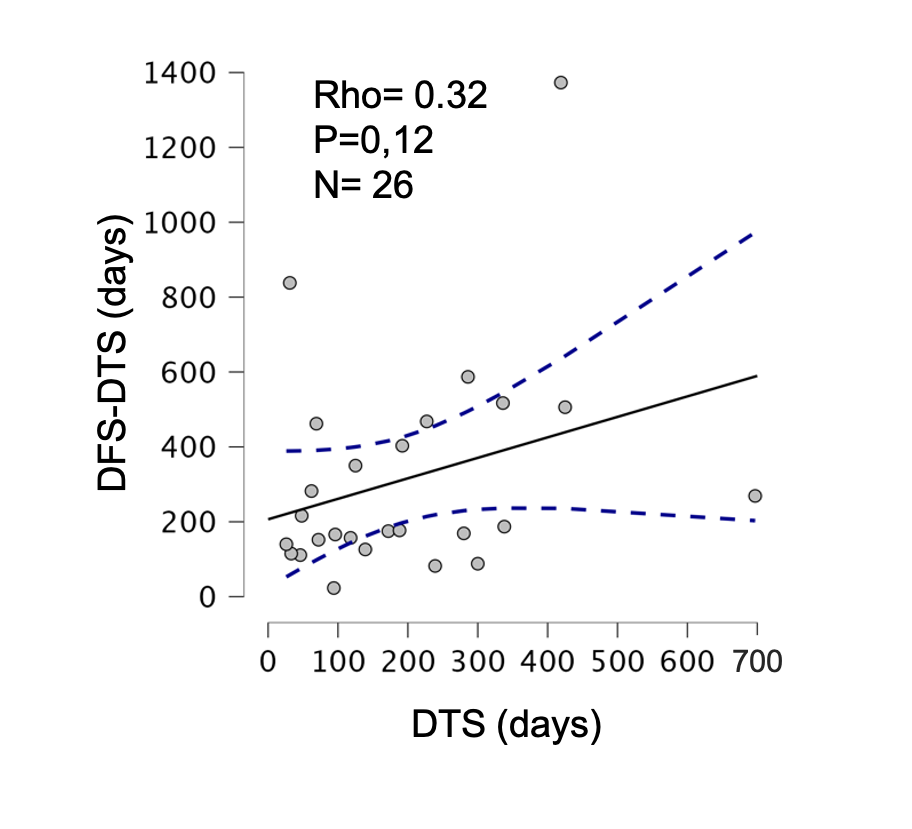

Supplement: oyad319_suppl_Supplementary_Figure [file oyad319_suppl_supplementary_figure.docx]
