# Supplementary material for: Hedgehog Inhibitors Beyond Clinical Complete Response in Basal Cell Carcinoma: Should I Stop or Should I Go?
Source: Oncologist. 2023 Dec 21;29(5):e699–707. doi: 10.1093/oncolo/oyad319 (PMC11067794; doi:10.1093/oncolo/oyad319)
Supplement: oyad319_suppl_Supplementary_Table [file oyad319_suppl_supplementary_table.docx]

**Supplementary Table 1** – The relationship between treatment duration and treatment discontinuation

|  | | **Days to complete response (DTCR, N=68)** | | **Days to vismodegib stop after cCR (DTS, N=61)*** | | **Total treatment days**  **(TTD, N=68)** | |
| --- | --- | --- | --- | --- | --- | --- | --- |
|  |  | **Median (range)** | **p-value** | **Median (range)** | **p-value** | **Median (range)** | **p-value** |
| **Vismodegib discontinuation** | Yes  (N=61)  No  (N=7) | 183.0 (56-595)  175.0 (140-506) | 0.686 | 125.0 (0-1018)  n/a | n/a | 367.0 (95-1106)  1530.0 (688-2112) | **<0.001**** |
| **Reason for vismodegib discontinuation (N=61)*** | Toxicity  (N=50)  Recurrence (N=11) | 176.0 (60-595)  195.0 (56-423) | 0.470 | 118.5 (0-1018)  322.0 (60-876) | **0.014**** | 326.5 (95-1106)  543.0 (217-932) | **0.020**** |

*Applicable to the 61 patients who discontinued vismodegib (7 patients received vismodegib until last follow-up)

**p<0.05 is considered significant. Values are reported in median (range)

*n/a, not applicable*
